# Computational Analysis of the Spatiotemporal Coordination of Polarized PI3K and Rac1 Activities in Micro-Patterned Live Cells

**DOI:** 10.1371/journal.pone.0021293

**Published:** 2011-06-27

**Authors:** Shaoying Lu, Tae-jin Kim, Chih-En Chen, Mingxing Ouyang, Jihye Seong, Xiaoling Liao, Yingxiao Wang

**Affiliations:** 1 Department of Bioengineering, University of Illinois at Urbana-Champaign, Champaign, Illinois, United States of America; 2 Neuroscience Program, University of Illinois at Urbana-Champaign, Champaign, Illinois, United States of America; 3 Beckman Institute for Advanced Science and Technology, University of Illinois at Urbana-Champaign, Champaign, Illinois, United States of America; 4 Department of Molecular and Integrative Physiology, Center of Biophysics and Computational Biology, University of Illinois at Urbana-Champaign, Champaign, Illinois, United States of America; 5 Institute of Biomaterials and Living Cell Imaging Technology, Chongqing University of Science and Technology, Chongqing, People's Republic of China; University of Birmingham, United Kingdom

## Abstract

Polarized molecular activities play important roles in guiding the cell toward persistent and directional migration. In this study, the polarized distributions of the activities of phosphatidylinositol 3-kinase (PI3K) and the Rac1 small GTPase were monitored using chimeric fluorescent proteins (FPs) in cells constrained on micro-patterned strips, with one end connecting to a neighboring cell (junction end) and the other end free of cell-cell contact (free end). The recorded spatiotemporal dynamics of the fluorescent intensity from different cells was scaled into a uniform coordinate system and applied to compute the molecular activity landscapes in space and time. The results revealed different polarization patterns of PI3K and Rac1 activity induced by the growth factor stimulation. The maximal intensity of different FPs, and the edge position and velocity at the free end were further quantified to analyze their correlation and decipher the underlying signaling sequence. The results suggest that the initiation of the edge extension occurred before the activation of PI3K, which led to a stable extension of the free end followed by the Rac1 activation. Therefore, the results support a concerted coordination of sequential signaling events and edge dynamics, underscoring the important roles played by PI3K activity at the free end in regulating the stable lamellipodia extension and cell migration. Meanwhile, the quantification methods and accompanying software developed can provide a convenient and powerful computational analysis platform for the study of spatiotemporal molecular distribution and hierarchy in live cells based on fluorescence images.

## Introduction

Directional migration plays an essential role in physiological and pathological conditions such as development, wound healing, and atherosclerosis [1]. Aberrant regulation of migration has been reported to indicate cancer metastasis [2]. Fibroblasts can also sense the spatial gradient of platelet derived growth factor (PDGF) and migrate to the wounded area [3], [4]. A typical migration procedure includes four steps: (1) extension of the lamellipodia; (2) formation of focal adhesions and stabilization of extension at the leading edge; (3) detachment of the focal adhesions at the tail; (4) contraction of the tail [1]. To coordinate these complex maneuvers for persistent migration, the cells need to sense the external cues, determine the migration direction and differentiate the molecular processes between the leading edge and the tail to achieve a polarity. Indeed, several signaling molecules display significant polarity during migration. Concentrated at the leading edge, the small GTPase Cdc42 plays important roles in sensing the direction and maintaining persistent migration in a microtubule-dependent manner [2], [5]. Integrin receptors, PI3K, and Rac1 also concentrate at the leading edge in active forms during directed migration [1]. The dynamic regulation and subcellular localization of these proteins further controls downstream signaling cascades in a polarized cell.

When a cell is exposed to a gradient of PDGF stimulation, PI3K is recruited by active PDGF receptors at the leading edge. Subsequently, PI3K catalyzes the phosphorylation of phosphatidylinositol 4,5-biphosphate (PIP2) and produces phosphatidylinositol 3,4,5-biphosphate (PIP3) [6], [7] to recruit and activate the Rac1 guanine nucleotide exchange factors (GEFs), such as Vav, Tiam-1, Swap-70, and Sos-1. These Rac1 GEFs can activate Rac1 and cause Rac1-dependent actin remodeling, which stimulates the protrusion at the leading edge [8]-[11]. Indeed, a strong spatial correlation between PIP3 and rapid membrane spreading has been reported [4], [12]. However, the detailed subcellular distribution of PI3K and Rac1 activities within the protrusion region as well as the relative temporal sequence of protrusion, PI3K and Rac1 activations remains unclear.

Previously, several experimental systems have been applied to study the process of polarization and migration: (1) micro-fluid chambers or micropipettes to introduce a gradient of chemo-attractant or extracellular matrix protein [13]–[16]; (2) wound healing assays to alter cell-cell interaction [17], [18]; (3) micropattern technology to manipulate the extracellular matrix environment [19]–[21]. Utilizing the micropattern technology and soft lithophotography fabrication, we recently developed a micropattern system to study cells seeded on extracellular matrix protein fibronectin (FN) strips separated by the non-adhesive copolymer, pluronic 127 [19]. In this system, the cells only adhere within the FN strips. When two cells form a junction on a FN strip, the cells are stably polarized with a free end capable of developing protrusions and a junction end connecting to a neighboring cell. As such, the polarized activities of lamellipodia, PI3K, and Rac1 can be well-controlled in a relatively one-dimensional space.

To efficiently and systematically obtain biologically relevant information from fluorescent live cell images, advanced computational analysis methods have been rapidly developed and applied [22], [23]. Some of the most prominent computational bio-imaging studies in migration include the analysis of speckles for studying the dynamics of actin filaments and the focal adhesion molecules [24], [25], the computational reconstruction of the stress map in gels from the displacement of the embedded fluorescent beads [26]–[28], and the recently developed computational multiplex method for studying the correlation of multiple signaling events by inter-related cross-correlation analysis [22], [29]. The importance of the computational multiplex method is underscored by a fundamental problem in fluorescence imaging, i.e., only a limited number of molecules can be monitored simultaneously in the same live cell. In an elegant study by Machacek et al, the computational multiplex method was used to show that RhoA was activated simultaneously with the initial extension of the leading edge at the local regions, while Rac1 and Cdc42 activation were both slightly delayed and located about 2 µm away behind the leading edge [29]. As such, the multiplex computing allowed the quantification of the signaling network with unprecedented spatiotemporal resolution.

In this paper, we developed an automated image analysis method for the quantification and visualization of polarized molecular events in a unified coordinate system in live cells seeded on micropattern strips. The maximal PI3K and Rac1 activity at the protrusion, and the position and velocity of the free end, were quantified and analyzed to provide a correlation between the signals in polarized cells under the stimulation of platelet-derived growth factor (PDGF). To implement these methods efficiently, we also developed an image analysis software package with a graphic user interface (GUI) and a programmer interface bridging the environment of MATLAB and Visual Basic to allow a broader usage of the computational analysis of polarized molecular signals by both experimental and computational biologists.

## Results

**Figure 1:**
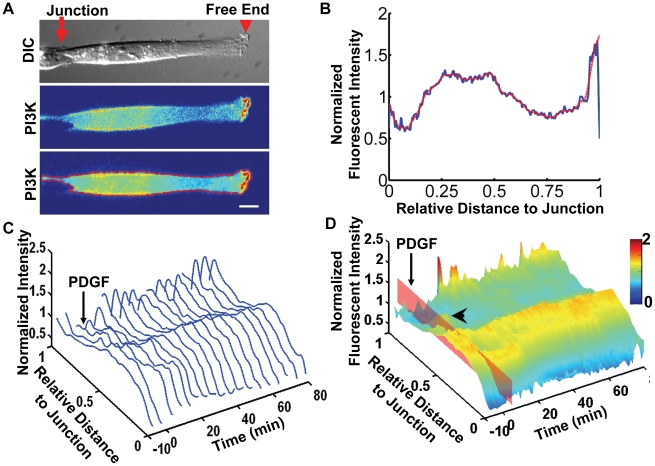
The quantification method and protein localization results. (**A**) The alignment and outline of a cell before quantification. Top panel: The DIC image of a cell with a junction connecting to a neighboring cell on a patterned FN strip and a free end capable of lamellipodial protrusion. Middle panel: The fluorescence image shows the intensity distribution of PH-Akt-GFP expressed in the cell of interest. Bottom panel: The cell was rotated and aligned along a horizontal direction, and the boundary of the cell calculated and overlaid in red with the fluorescence intensity image. Scale bar: 10 µm. (**B**) The normalized fluorescence intensity of PH-Akt-GFP plotted against the relative distance to the junction end with the blue and red lines representing the raw and smoothed data, respectively. (**C**) The sequence of the normalized fluorescence intensity curves arranged along the time axis with a 3D view. The PDGF simulation was applied at 0 min. (**D**) The 3D intensity surface of PI3K activity landscape was color-coded by the fluorescence intensity values and visualized as a function of time and distance to the junction end, based on the data in **(C)**. The time of PDGF stimulation is indicated by the red plane. A transient secondary peak can be observed between the cell body and the free end (arrowhead). The spatiotemporal dynamics of PI3K activity in this cell is also shown in Movie S1.

The mouse embryonic fibroblasts (MEFs) were confined to adhere on fibronectin-coated strips, with a stable junction end connecting to a neighboring cell and a free end without cell-cell contacts (Fig. 1A). The fluorescent intensity of probes expressed in these MEFs at subcellular locations can be quantified as a function of the distance to the junction. As shown in Figure 1A, the cell was transfected with PH-Akt-GFP, where the PH domain of Akt was fused to a green fluorescent protein (GFP). Since PH-Akt-GFP binds to PIP3, a product of active PI3K, the subcellular distribution of PH-Akt-GFP can represent the localization of active PI3K [30]. To quantify the subcellular fluorescence distribution, the fluorescent image of the cell was rotated such that the direction of the FN strip was aligned to the x-axis, with the free end pointing to the right (Figure 1A). The fluorescent intensity image was then segmented using the Otsu's method with a chosen threshold [31], so that the intensity image was converted into a binary mask image with values outside the cell set to zero. The fluorescent intensity of PH-Akt-GFP within the mask was averaged in the y-direction and plotted against the normalized distance to the junction (Figures 1A and 1B). In order to investigate and compare the fluorescent intensity curves from the same cell at different time points or those from different cells with various shapes under the same scale, the length of each cell body was normalized to unity. The total fluorescent intensity along the cell body was also normalized to unity, to correct for the artificial variation of fluorescent intensity caused by different levels of protein expression or a shift of focus. The normalized curve was further smoothed by a robust and locally weighted quadratic regression method [32], [33] to eliminate the imaging noise (Figure 1B, for details see the Materials and Methods section). To obtain the spatiotemporal landscape of the PI3K activation, the normalized and smoothed curves of PH-Akt-GFP intensity were aligned along the time axis to generate a surface plot (Figures 1C and 1D).

The surface plot of the fluorescence intensity represents PI3K activity as a function of time and the relative distance to the junction (Figure 1D). The result clearly indicates that active PI3K increased at the free end upon uniform PDGF stimulation, possibly due to the subcellular recruitment or activation of PDGFR (Figure 1D) [9], [34]. The fluorescence intensity near the nuclear regions remained high before and after PDGF stimulation, probably reflecting the 3D morphology of the cell. In the fluorescent intensity landscape, there was a second peak that formed between the cell body and the free end soon after addition of PDGF (Figure 1D), possibly representing the fluorescent probes trapped in an exocytosis recycling route being transported to the plasma membrane [35]. The PDGF stimulation also induced both the increase of PI3K activity and lamellipodium edge extension at the free end in a dynamic and wave-like fashion (Figure 1D and Movie S1). The high peaks of PH-Akt-GFP intensity appeared to be associated with the ruffling events leading to a significant edge extension. This peak PH-Akt-GFP fluorescent intensity mainly resulted from locally concentrated PI3K activity at the ruffles, but not from the physical property of the ruffles in trapping more fluorescent probes. Indeed, the ratio between the intensity of PH-Akt-GFP and that of a membrane-bound Lyn-mCherry fluorescent probe [36] was significantly higher at the ruffles and the protrusion regions (Figure S1).

**Figure 2:**
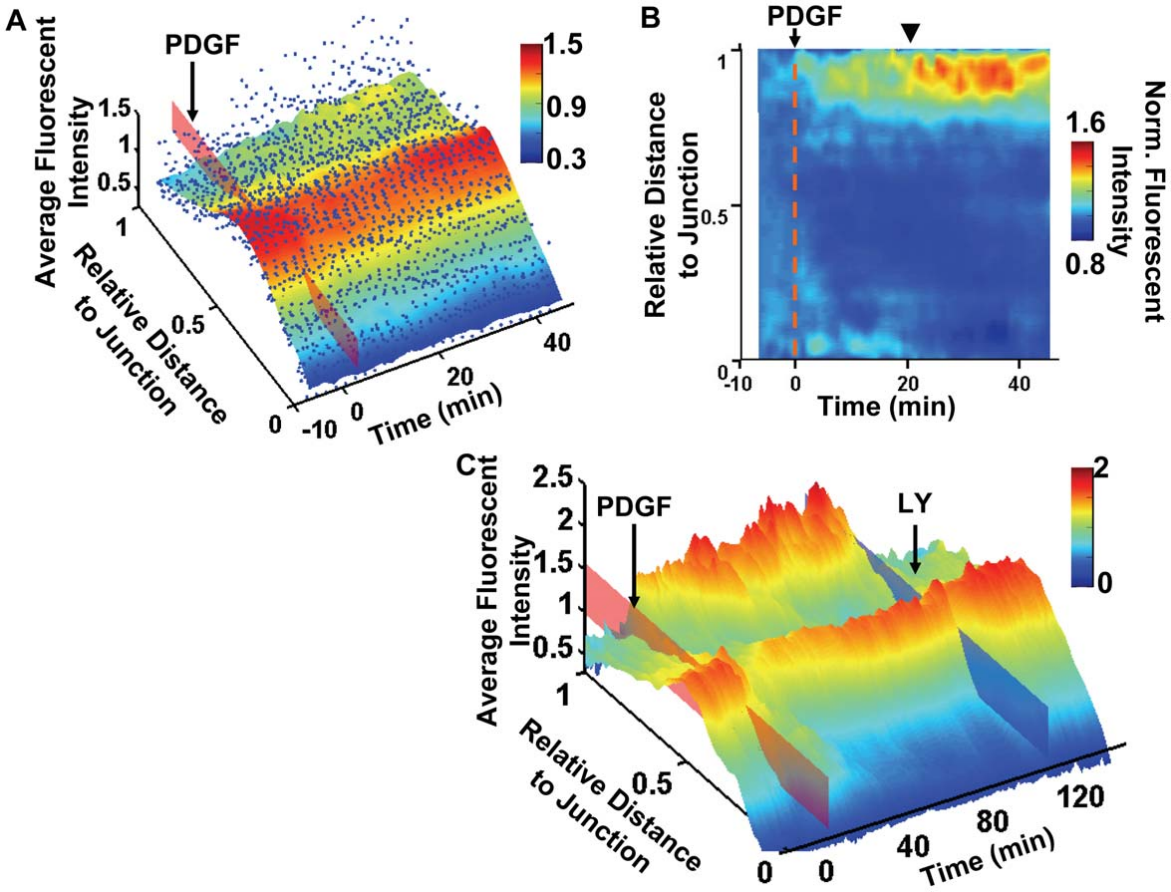
The averaged and normalized PI3K activity landscapes. (**A**) The fluorescence intensity of PI3K averaged across multiple cells is shown as a function of time and distance, color-coded by the fluorescence intensity values. The blue dots represent the intensity data points from multiple cells (*n = 14*) used for computing the average intensity. The time of PDGF stimulation is indicated by the red plane. A 3D rotational view of the average landscape surface with the data points is shown in Movie S2. (**B**) The average surface is normalized by the initial intensity curve before PDGF stimulation and shown with a 2D view. After PDGF stimulation (dotted red line), the normalized fluorescence intensity increased near the free end while decreasing near the junction and the perinuclear/nucleus regions. This normalized fluorescence intensity peaked at 21 min after PDGF at the free end, as indicated by the arrow head. (**C**) The PI3K activity landscape in an MEF treated with PDGF (0 min, red plane) prior to LY application (∼120 min, blue plane). The spatiotemporal dynamics of PI3K activity in the cell under PDGF stimulation followed by LY inhibition is shown in Movie S3.

To compare the data from multiple cells (*n = 14*), the spatiotemporal landscapes of PI3K activity from all the cells were collected to compute an average surface in three steps (Figure 2A and Movie S2). First, the fluorescence intensity values of different PI3K landscapes were interpolated and calculated at the same coordinates of time and distance. Second, at each time point, the intensity curves from all cells were combined to compute an average curve with robust and locally weighted quadratic regression (for details see the Materials and Methods section) [32], [33]. Third, the average curves at different time points were aligned to visualize the average surface (Figure 2A). This result confirmed that PI3K activity was slightly polarized before PDGF stimulation [4] and showed that uniform PDGF stimulation can further enhance the polarity of PI3K activity in patterned cells. To eliminate the noise originated from the cell morphology and perinuclear trapping of fluorescent probes, a normalized surface was calculated by dividing the intensity curve at each time point against the curve before PDGF stimulation. This normalization procedure can eliminate the basal-level polarity and highlight the changes in the fluorescent distribution landscape induced by PDGF stimulation. With this normalized PI3K activity landscape, it becomes clear that active PI3K increased at the free end after PDGF stimulation, which was accompanied by a concurrent decrease near the nucleus and junction end to result in a more polarized landscape (Figure 2B). In fact, the polarization of PI3K activity increased immediately following the PDGF stimulation and peaked after ∼21 min. Therefore, both the average and normalized landscapes supported the notion that PDGF can induce an enhanced polarization of PI3K activity in micropatterned cells. It is of note that on the average and normalized landscapes, the wave-like activation pattern of PI3K activity at the free end observed in a single cell became less apparent, possibly because of the different oscillation frequencies among different cells. A specific inhibitor of PI3K, LY294002 (LY), completely abolished the accumulation of PH-Akt-GFP at the free end and eliminated the polarity of Ph-Akt-GFP (Figure 2C and Movie S3). In contrast, ML-7, an inhibitor of myosin light chain kinase (MLCK) to suppress the actomyosin contractility, had no effect on the polarity of PI3K activity (data not shown). These control experiments confirmed that the PH-Akt-GFP probe can specifically detect and monitor the localization of active PI3K.

**Figure 3:**
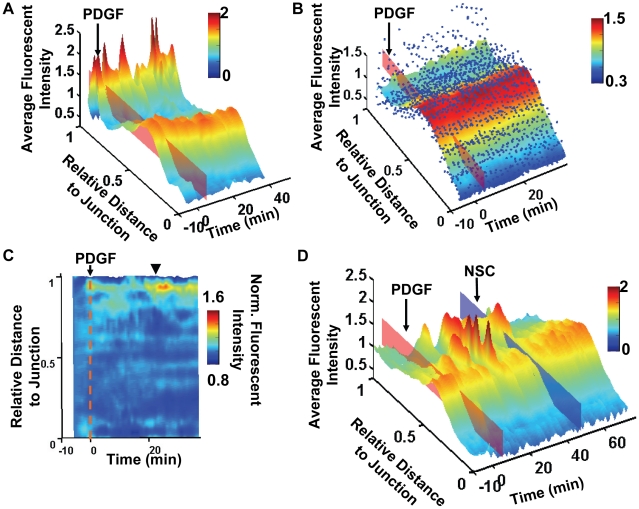
The Rac1 activity landscapes. (**A**) The fluorescence intensity surface of PAK-PBD-YFP representing the Rac1 activity of a representative MEF before and after PDGF stimulation (red plane) is shown as a function of time and the relative distance to the junction. The surface is color-coded by the value of fluorescence intensity. The spatiotemporal dynamics of Rac1 activity in this cell is shown in Movie S4. (**B**) The Rac1 activity landscape surface is computed by averaging across multiple cells. The blue dots represent the fluorescence intensity data points from multiple cells (*n = 12*). A 3D rotational view of this average landscape surface with the data points is shown in Movie S5. (**C**) The average Rac1 activity landscape is normalized by the initial intensity curve before PDGF stimulation and shown in a 2D view. After PDGF (the dotted red line), the relative Rac1 activity increased moderately near the free end while decreasing near the nucleus and the junction. This relative increase of Rac1 activity peaked at ∼23 min after PDGF, as indicated by the arrow head. (**D**) The Rac1 activity landscape of an MEF treated with PDGF (0 min, red plane) prior to NSC application (∼40 min, blue plane) is shown as a function of time and the distance to the junction.

We then applied PAK-PBD-YFP, another fluorescent probe containing a PAK domain capable of binding to active Rac1, to monitor the distribution of active Rac1 which cooperates with PI3K during cell migration [37]. As in the case of PH-Akt-GFP, both the fluorescence intensity of PAK-PBD-YFP and the lamellipodia at the free end displayed wave-like dynamics in individual cells upon PDGF stimulation (Figure 3A and Movie S4). Interestingly, the Rac1 activity landscape either generated from a representative cell or averaged over multiple cells (*n = 12*) revealed that the basal Rac1 activity was high at the free end, which can be further enhanced by PDGF stimulation (Figures 3A and 3B, Movie S5). While the normalization procedure against the curve before PDGF masked the initial polarity, the Rac1 activity landscape after this normalization revealed that PDGF induced an immediate increase of Rac1 polarity near the free end, which peaked at ∼23 min (Figure 3C). Control experiments confirmed the specificity of the PAK-PBD-YFP probe since the reported Rac1 activity at the free end was blocked by a specific Rac1 inhibitor NSC (Figure 3D) [38], [39]. In addition, the Rac1 activity was partially blocked by the PI3K inhibitor LY (Figure S3A), but not by the MLC inhibitor ML-7 (data not shown). Rac1 inhibitor NSC also reduced PDGF-induced PI3K activity at the free end and blocked the front edge protrusion with some time delay (Figure S3B). This is consistent with the positive feedback loop established by PI3K and Rac1 activities in migrating cells via GEF and superoxide [9], [10], [40], [41]. It is of note that PDGF induced a sustained PI3K activation covering about 20% of the cell length near the free end, while a weaker Rac1 activation was restricted to a very narrow region (<10% of the cell length, Figures 2 and 3). These results also suggest possibly distinct regulation mechanisms of PI3K and Rac1 at these subcellular regions.

The wave-like molecular activity of PI3K and Rac1 at the free end of the cells appeared correlated to the periodic extension and retraction of the lamellipodium. We hence quantified the time course and utilized the multiplex analysis method to compute the cross-correlation between different molecular activities (represented by the maximal fluorescent intensity of the molecular probes Ph-Akt-GFP or PAK-PBD-YFP) and morphological changes (edge position or extension/retraction velocity) at the free end. Our analysis was based on the following assumption: (1) at the free end, the increase of edge position is considered to represent the extension of the lamellipodia, while the increase of velocity represents the initiation of new lamellipodia extension; (2) both PI3K and Rac1 activities are expected to positively regulate the lamellipodia extension. The cross-correlation analysis also required that the cells have significant lamellipodia dynamics, so only the cells with more than 2 µm of extension at the free end after PDGF stimulation were included in the analysis.

**Figure 4:**
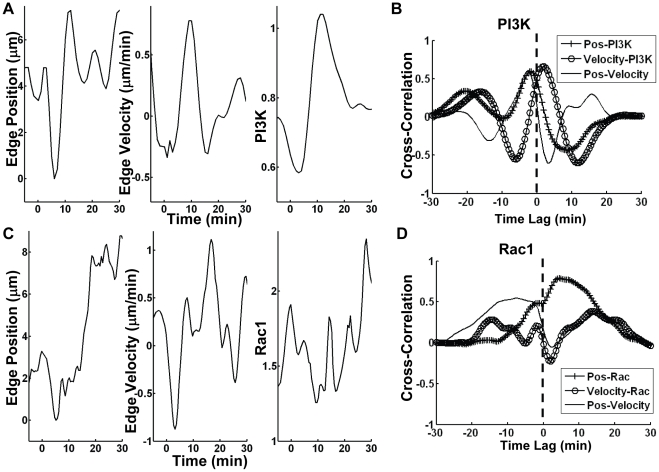
The cross-correlation (CC) curves. (**A** and **C**) The time course of the edge position (left), the edge velocity (middle) and the maximal fluorescence intensity (right) at the free end is shown in a representative cell expressing (**A**) PH-Akt-GFP or (**C**) PAK-PBD-YFP probe before and after PDGF stimulation (0 min). (**B**) The CC curves of Position-PI3K (Pos-PI3K) (+), Velocity-PI3K (o), and Position-Velocity (Pos-Velocity) (solid line) from the cell in (**A**), are plotted against the time lag. (**D**) The CC curves of Position-Rac (Pos-Rac) (+), Velocity-Rac (o), and Position-Velocity (Pos-Velocity) (solid line) from the cell in panel (**C**) are plotted against the time lag.

Because the movement of the cells was constrained to the approximately 1-D space on the FN strips, the furthest pixel of the cell away from the junction end along the x-axis was identified and its position converted to the edge position of the free end (Figure S2). The central difference of the edge position was then computed to represent the edge velocity of the free end[42]. Meanwhile, the maximal fluorescence intensity of probes was obtained by finding the maximal value of the intensity within the free end region (10% of cell length, Figure S2). Thereafter, the position and velocity of the edge and the maximal fluorescence intensity of different probes at the free end were plotted vs. time for representative cells expressing PH-Akt-GFP (Figure 4A) and PAK-PBD-YFP (Figure 4C). The time courses of edge position, velocity, and fluorescence intensities representing either PI3K or Rac1 activity at the free end all showed dynamic trajectories.

To investigate the signaling sequence of these events: (1) the change of the edge position, (2) velocity and (3) front fluorescence intensity, the cross-correlation (CC) curves between each pair of signals in Figures 4A and 4C were calculated for two representative cells and shown in Figures 4B and 4D respectively. Briefly, a peak value of the CC curve between two signals A and B with a positive time lag indicates that signal A occurs earlier than signal B [22], [29]. For example, in a representative cell expressing the Ph-Akt-GFP probe, the Velocity-PI3K CC curve had a peak with a positive time lag, indicating that the PDGF-induced extension of lamellipodia initialized before the PI3K activation (Figure 4B). In contrast, the peak of the Position-PI3K CC curve occurred at a negative time lag, indicating that the PI3K activity increases before the persistent and stable lamellipodia extension at the free end (Figure 4B). In another representative cell expressing the PAK-PBD-YFP probe, the peaks of both the Position- and Velocity-Rac1 CC curves occurred with positive time lags (Figure 4D), suggesting that the PDGF-induced Rac1 activation occurred after the increase of velocity and edge position of the free end. These results suggest that it is possible to study the signaling sequence of the initiation of lamellipodia extension, PI3K activation, the persistent and stable lamellipodia extension, and Rac1 activation based on the data from different groups of cells.

To examine the accuracy of our cross-correlation analysis models, the Position-Velocity CC curve was also computed and plotted. This CC curve showed a peak with a negative time lag for both cells (Figure 4B and 4D), consistent with the physical understanding that the initialization of lamellipodia extension (the edge velocity) occurs before the persistent/stable extension of the lamellipodia (the edge position). Therefore, our CC analysis can be applied to study the temporal relationship between the local molecular activities and the morphological/biophysical changes of the cell edge.

**Figure 5:**
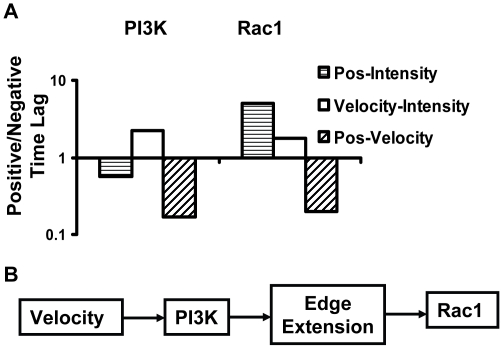
The time sequence of molecular events. (**A**) Bar graphs represent the ratios of cell numbers with positive vs. negative time lags in Position-Intensity, Velocity-Intensity and Position-Velocity CC curves for the cells expressing the fluorescent probes measuring the PI3K and Rac1 activities, as indicated. (**B**) A proposed model depicting the sequence of molecular events based on the results from (**A**).

To quantitatively analyze the activation sequence of PI3K and Rac1 in a group of cells under PDGF stimulation, the CC peak values and peak time lag of multiple cells were collected and compared (PI3K: *n = 14*; Rac1: *n = 12*). Due to the cell variability, we counted the number of cells with positive and negative time lags for each CC pair of PI3K and Rac1 activity, including position-molecular activity, velocity-molecular activity, and position-velocity. The ratio of the cell counts with positive and negative lags was then computed for each CC pair, between two chosen signals A and B. A ratio value greater than 1 would indicate that signal A occurred before signal B in majority of the cell population. Conversely, a ratio value less than 1 would indicate that signal A occurred after signal B. Our results hence suggest that in a majority of the cells, the velocity of the free end and therefore the initiation of lamellipodia extension increased upon PDGF stimulation before the local PI3K activation, which was followed by stable edge extension (Figure 5). This is also confirmed by the observation that the inhibition of PI3K activity by LY halted the extension of the free end and caused it to retract (Movie S3). Interestingly, the increase of both free-end velocity and edge-position occurred before the Rac1 activity in the majority of the cells examined, as evidenced by the larger number of cells with positive lags comparing to those with negative lags (Figure 5A). Meanwhile Rac1 inhibitor partially blocked PI3K activity and inhibited the protrusion of the free end (Figure S3). These results suggest that Rac1 might be dispensable for both the initiation of the free-end extension and the initiation of the PI3K activation induced by PDGF (Figure 5B). This is consistent with previous reports that (1) PI3K can activate Rac GEFs and subsequently Rac1 upon stimulation [9], [10]; (2) the leading edge extension of a dynamic cell occurred before Rac1 activation [29]. As a control analysis, we confirmed that changing the discretization algorithms in calculating the velocity (from the central difference to the forward or backward difference) did not affect the results and conclusions. In addition, the cells expressing different fluorescence probes had similar dynamic behavior at the free end (Figure S4). Therefore, our results can reliably and accurately support the notion that the initialization of free-end extension occurs before PI3K, which is followed by persistent free-end extension and subsequently Rac1 activation (Figure 5B).

## Discussion

In this paper, we developed a computational method to analyze and visualize the subcellular distribution of molecular activity detected by fluorescent probes. This analysis method was applied to quantify the PDGF-induced PI3K and Rac1 activation pattern in polarized MEFs on micro-patterned strips. We further applied a multiplex computational method to analyze the sequential signaling cascades of PI3K and Rac1 activities, as well as the edge dynamics [29]. The results suggest that, at the free end after PDGF stimulation, the initiation of the edge extension precedes PI3K activation, which leads to the stable edge extension followed by Rac1 activation. A software package was also developed with an image analysis module to perform the fluorescence landscape quantification and visualization, and with a graphic user interface module to allow the convenient preprocessing of fluorescence images.

Since the normalized intensity curves were plotted against the normalized distance to the junction, this approach allowed the normalized cell length and total fluorescence intensity to remain constant during the time course of an experiment and among different cells. Therefore, the computational analysis method provided a uniform framework for the quantification and analysis of the subcellular distribution of molecular activities from multiple cells with different and changing shapes. With this unified framework, the molecular activity landscape in time and space can also be constructed to visualize the dynamic patterns of the polarized molecular distribution in a cell. The activity landscapes from multiple cells can further be assembled together to compute an average activity landscape. Therefore, our computational analysis and visualization method can provide a powerful tool for the integrative analysis of molecular activity monitored by fluorescent probes in live cells.

The subcellular PI3K activity in fibroblasts as reported by PH-Akt-GFP fluorescence intensity has previously been extensively studied by the Haugh group using live-cell imaging and computational analysis [4], [43]-[46]. They reported that polarized PI3K activity can be further enhanced by a PDGF gradient but not uniform PDGF in free-migrating fibroblasts [4]. Interestingly, in polarized fibroblasts with a single cell-cell junction on micro-patterned strips, a uniform PDGF stimulation can also enhance the PI3K polarity. This result highlights the importance of micro-environment to cell migration. In addition, it has been suggested by Haugh et al. that the PH-Akt-GFP intensity represents a combined effect of PI3K activation via PDGFR at the top surface membrane, PIP3 diffusion and the phosphatase-catalyzed deactivation at the center of the cell/glass interface, as indicated by a consistent axial gradient of PH-Akt intensity after uniform PDGF stimulation [4], [43], [44], [46]. Our results also support this finding as shown in the intensity profile of PH-Akt-GFP and the ratio profile between PH-Akt-GFP and Lyn-mCherry (Figs. 1A and S1).

In analyzing the cross-correlation (CC) curves, we assumed a positive correlation between the activity of PI3K/Rac1 and the edge dynamics based on previous publications, in searching for the location of peaks on the CC curves. The CC results showed that there is a significant heterogeneity among different cells since the CC peak time lag from different cells had varied values. This time-lag difference between the PI3K/Rac1 activity and the edge position/speed at the free end may be attributed to relatively unstable events in a single cell involved in the expansion of the edge and actin polymerization. For example, the expansion of the leading edge of a migrating cell may be correlated to the breaking point of the membrane curvature due to an accumulated mechanical stress after a sustained actin polymerization [47], but not directly correlated to the actin polymerization event itself. Nevertheless, the ratio of cell numbers with negative and positive time lags should capture and provide the main characteristics of a whole cell population. Indeed, our ratio analysis results support the notion that Rac1 and PI3K activations follow the initiation of edge extension upon PDGF stimulation. This is consistent with recent reports that the initial lamellipodia extension in quiescent cells is relatively independent of Rac1 activity, but more correlated to the RhoA-mDia pathway [29]. It has been reported that the small GTPase RhoA is active at the leading edge and coordinates the initialization of the migration process [29], [48], [49]. RhoA can activate mDia which stabilizes the actin filaments by serving as a “leaking cap” at the barbed end to ensure the continuous assembly of linear actin filaments [50]-[52]. In addition, it has been recently reported that the actin filaments at the tip of the migrating cells are mostly unbranched [53], [54], suggesting that the extension of the actin-filaments at the migration front may be independent of the Rac1-Arp2/3 pathway.

In addition to the time lag information derived from the CC curves, the peak values of the CC curves provide information on the level of correlation in the data. The peak value of the CC curve between two signals can vary from 1 (the two signals are identical in profile) to 0 (the two signals are completely not correlated). Interestingly, the average peak values of all the cross-correlation curves are greater than 0.4, indicating a significant correlation between all the quantities studied (Figure S5). It is of note that the PI3K activity was in general more strongly correlated with the edge dynamics at the free end than the Rac1 activity (Figure S5). This variation in the CC peak values possibly represents a better correlation between the free end dynamics and the PI3K activity than that of the Rac1 activity. This result is also consistent with previous analysis indicating that PI3K activity drives spreading and random migration of fibroblasts [12], [55].

The software package developed during this study is specifically designed to examine and analyze images of live cells. Indeed, this software package has been applied to successfully quantify and analyze the polarized signals of integrin, PI3K, Rac1, MLCK and actin (M. Ouyang et al. manuscript in submission). With the graphic user interface in Visual Basic and a flexible programmer's interface in MATLAB (see Materials and Methods), it is expected that this software can provide a powerful, convenient, and general tool for biologists to analyze live cell images and develop customized programs based on this platform.

## Materials and Methods

### Cell Culture, Transient Transfection, and Reagents

Mouse embryonic fibroblasts (MEFs) (from ATCC) were cultured in Dulbecco's modified Eagle's medium (DMEM) supplemented with 10% fetal bovine serum, 2 mM L-glutamine, 100 unit/ml penicillin, 100 µg/ml streptomycin, and 1 mM sodium pyruvate (GIBCO BRL) in a humidified 95% air, 5% CO_2_ incubator at 37°C before transfection. Different DNA plasmids were transfected into cells by using Lipofectamine 2000 according to the protocol from the vendor (Invitrogen). The transfection condition was optimized for MEFs, with 3 µg of DNA plasmids for each 35 mm cell culture dish. If the cell density was high (e.g.>80% confluency), 4 µg of DNA plasmids were applied for each dish. Cells expressing various exogenous proteins were starved in cell culture medium with 0.5% FBS for 36–48 hr before passing on fibronectin-coated micropatterned glass surface for 2–6 hr prior to imaging. To avoid the detrimental effects of trypsin on cell migration when passing cells, cells were detached by a mild 4 mM EDTA (pH 7.4, incubation at 37°C for 8–10 min) in PBS.

Recombinant rat platelet-derived growth factor-BB (PDGF-BB 50 ng/ml) and myosin light chain kinase (MLCK) inhibitor ML-7 (5 µM) were purchased from Sigma-Aldrich. PI3K inhibitor LY294002 (10 µM) and Rac1 inhibitor NSC23766 (50 µM) were purchased from Calbiochem, EMD Bioscience. In all experiments, the PDGF-BB solution was added into and immediately mixed with the medium to ensure that the cells were stimulated with a uniform concentration of the growth factor.

### Microscope Image Acquisition

During imaging, cells were cultured in cover glass bottom dishes (Cell E&G) and maintained in CO_2_-independent medium (Gibco BRL) without serum at 37°C. Images were collected by a Zeiss Axiovert inverted microscope equipped with 40x objective (NA:1.30; resolution: 250 nm) and a cooled charge-coupled device camera (Cascade 512B; Photometrics) using the MetaFluor 6.2 software (Universal Imaging). The parameters of dichroic mirrors, excitation and emission filters for different fluorescent proteins were described previously [56]. The fluorescence intensity images of different probes were represented by pseudo colors, with cold and hot colors representing low and high intensities, respectively.

### Image Analysis Methods and Software

**Figure 6:**
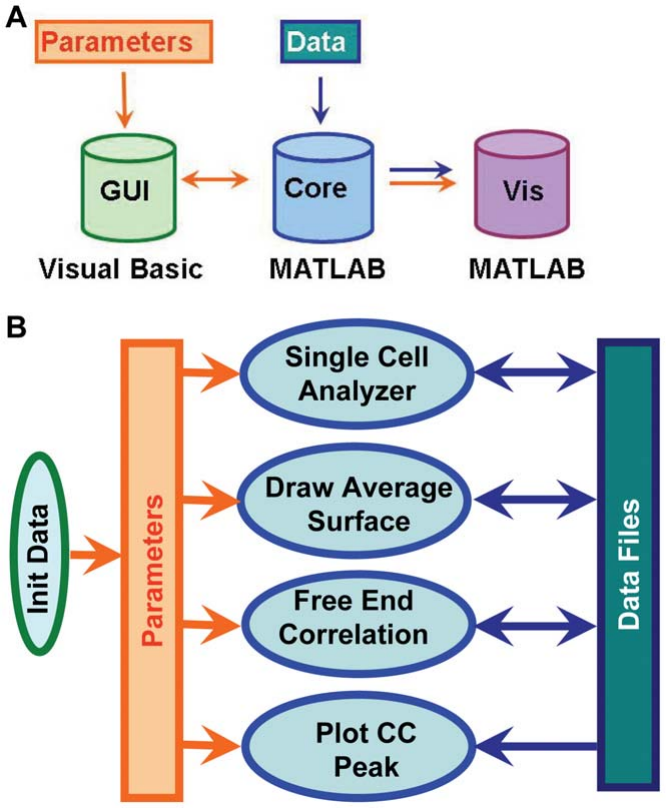
Software structure and data flow patterns. (**A**) The graphic user interface module implements programs in Visual Basic interfaced with MATLAB for data processing and analysis. (**B**) In the core image analysis module, the init_data function is used to initialize parameters such as the location of image files, segmentation threshold and rotation angle for image processing. The initialized parameters are used as input for the image analysis functions including single_cell_analyzer, draw_average_surface, free_end_correlation and plot_cc_peak, which also allow the input of image data files and the output to additional data file. In both (**A**) and (**B**), the orange-colored arrows represent the data flow of parameters, and the dark-blue-colored arrows represent the input/output of data files.

To analyze the experimental data conveniently, we developed and implemented a software package, *fluocell*, including programs in MATLAB and Visual Basic (VB). To establish a convenient user interface, we have implemented a modulated design and object-oriented programming methods. As illustrated in Figure 6, the software contains two modules: a pre-processing module with a graphic user interface in VB, and an image analysis module with a programmer's interface in MATLAB (Figure 6A). The first module allows users to visually examine the fluorescence intensity data and pre-process the data by filtering, background-subtraction and cropping (Figures S6 and S7). It also allows the interactive selection of the rotation angle and segmentation threshold for computing the mask of the fluorescent cell. A single threshold can be selected and applied to all the images in the video sequence of a cell to allow the automation. The second module contains a computational analysis core with a programming interface for users (Figure 6B). This module allows the sequential execution of functions such as single cell analysis, average surface computing, and cross-correlation curve calculation and visualization (Figure 6B). Both modules can be installed and used in the Windows operating system running MATLAB.

Because of the large amount of data and intensive analysis nature of our project, a balanced strategy between data storage and computational efficiency was adopted. For example, the sequence of background-subtracted images was not stored. Instead, a single mask outline of the selected background region was stored for the whole image sequence to allow the computation of the background-subtracted images in real time. Meanwhile, intermediate images/results after the execution of a computationally demanding function were stored, which can be conveniently re-loaded if further modification and analysis are required. These strategies led to the high convenience, flexibility, and efficiency of our software package in handling both the large amount of data and computationally demanding tasks of live cell imaging analysis.

All the fluorescence images were background-subtracted before image quantification and analysis. The boundary outlining the mask of the cell body was calculated utilizing the MATLAB (Mathworks; Natick, MA) function *smooth* with the ‘lowess’ option using a window size of 3 pixels (1.17 µm with a 40x objective) while the intensity distribution curves and surfaces in space were calculated using the same function with the ‘rloess’ option and moving window size of 10% of cell length. The curves representing the time courses of the edge position, velocity, and fluorescence intensity at the free end were also smoothed with the ‘rloess’ option using a moving window size of 20 min. The time courses before and after PDGF stimulation (from −5 to 30 min) were applied to calculate the CC curves (Figure 4). In this case, it was important to include the time course before PDGF to capture the changes caused by the growth factor. The interface between Visual Basic (Microsoft Corporation; Redmond, WA) and MATLAB was provided by the automated server within MATLAB. The source code is published via GoogleCode (http://code.google.com/p/polarityanalysis/downloads/list).

## Supporting Information

Figure S1 **Ratiometric Comparison of the PH-Akt-GFP and Lyn-mCherry localization.** The PH-Akt-GFP/Lyn-mCherry ratio image sequence is shown for a polarized cell on pattern, before and after PDGF stimulation. The GFP/mCherry ratio had a relatively even profile before stimulation. After stimulation, the ratio image showed a small value (blue) at the center of cell body, a intermediate value (green) at two lateral sides of the patterned region and a large value (red) in membrane ruffles (middle panel, white arrow) and the protrusion region (bottom panel, white arrow head), indicating that PH-Akt-GFP had a more significant presence in the ruffles and protrusion than Lyn-mCherry. Scale bar: 10 µm.
(TIF)

Figure S2 **The edge position at the free end.** After the fluorescent image of a cell is rotated and aligned along the x-axis with its junction to a neighboring cell at the left side, the edge position of the free end (red arrow) is calculated based on the maximal x-pixel number of the detected cell mask (outlined in red). The free end region is chosen as the 1/10 of cell length at the free end, as outlined in the white and dashed rectangle.
(TIF)

Figure S3 **The effect of inhibitors. (A)** The Rac1 activity landscape of an MEF treated with PDGF (0 min, red plane) prior to PI3K inhibitor (LY) application (∼80 min, blue plane) is shown as a function of time and the distance to the junction. **(B)** The PI3K activity landscape of an MEF treated with PDGF (0 min, red plane) prior to Rac1 inhibitor (NSC) application (∼50 min, blue plane) is shown as a function of time and the distance to the junction.
(TIF)

Figure S4 **The expression of different fluorescent probes did not affect the front-edge dynamics. (A)** and **(B)**, the smoothed time courses of the front edge position in the cells with PI3K or Rac1 probes respectively, with each line representing one cell; **(C)** and **(D)**, the smoothed time courses of the front edge velocity in the cells with PI3K or Rac1 probes respectively, with each line representing one cell. The shaded regions indicate negative velocity.
(TIF)

Figure S5 **The cross-correlation peak values.** The mean and standard error of means (SEM) of the peak values of the cross-correlation curves are shown for PI3K and Rac1 activities.
(TIF)

Figure S6 **The graphic user interface (GUI).** The GUI was implemented in Visual Basic. It allows the users to display, filter, rotate and crop images, and adjust the rotation angle and threshold value of imaging segmentation for each cell.
(TIF)

Figure S7 **Image pre-processing with GUI.** Left: the raw data image shown in pseudo color. Right: the raw image was processed by a medium filter with a window size of 3×3 pixels, background-subtracted, intensity-scaled, and rotated so that the free end of the cell was aligned along the positive direction of the x-axis. The image segmentation threshold was also selected and the boundary of the cell mask calculated and shown in solid red. The orange block on the lower left corner of the image highlights the area where the background signal was calculated for subtraction.
(TIF)

Movie S1 **The distribution of PI3K activity in a representative MEF under PDGF stimulation.** The fluorescent images representing the subcellular distribution of PI3K activity were overlaid on top of the DIC images of the MEF cells under PDGF stimulation. The MEFs were seeded on FN strips in the horizontal direction. The cell expressing PH-Akt-GFP was highlighted in pseudo-color among other cells without PH-Akt-GFP expression. PDGF stimulation induced a significant increase of free end dynamics accompanied by an increase of PH-Akt-GFP fluorescence (PI3K activity).
(AVI)

Movie S2 **The PI3K activity landscape surface averaged across multiple cells.** The averaged fluorescence intensities are pseudo-color-encoded with hot and cold color representing the low and high fluorescence intensities, respectively. A rotational view of the average surface was shown with raw data points (blue dots) from all the cells.
(AVI)

Movie S3 **The distribution of PI3K activity in an MEF under PDGF stimulation followed by LY inhibition.** The fluorescence images representing the subcellular distribution of PI3K activity were overlaid on the DIC images of the MEFs. PDGF stimulation induced a significant increase of free end dynamics accompanied by an increase of PH-Akt-GFP fluorescence (PI3K activity). Adding LY inhibited PI3K activity at the free end and caused its retraction within 10 min.
(AVI)

Movie S4 **The distribution of Rac1 activity in a representative MEF under PDGF stimulation.** The fluorescence images representing the subcellular distribution of Rac1 activity were overlaid on the DIC images of the MEF cells. The cell expressing PAK-PBD-YFP was highlighted among other cells without fluorescence. Before and after the PDGF stimulation, the free end of the cell appeared dynamic with strong PAK-PBD-YFP fluorescence representing high Rac1 activity at the leading edge of the free end.
(AVI)

Movie S5 **The Rac1 activity landscape surface averaged across multiple cells.** The average landscape surface was calculated to summarize the data from multiple cells. A rotational view of the average surface was shown with raw data points (blue dots) from all the cells.
(AVI)
