# Supplementary figures and images for: Computational Analysis of the Spatiotemporal Coordination of Polarized PI3K and Rac1 Activities in Micro-Patterned Live Cells
Source: PLoS One. 2011 Jun 27;6(6):e21293. doi: 10.1371/journal.pone.0021293 (PMC3124492; doi:10.1371/journal.pone.0021293)

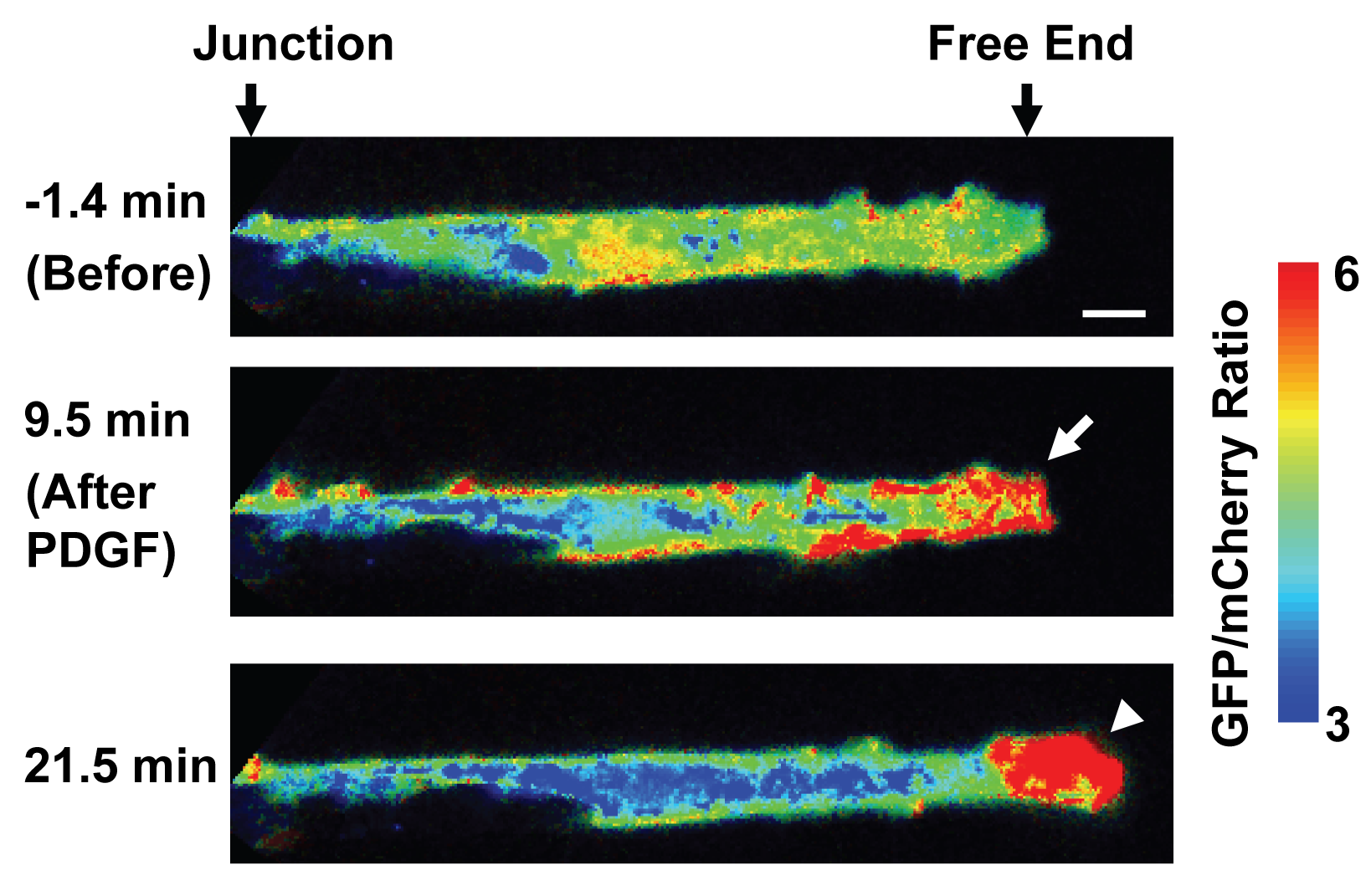

Supplement: Figure S1 — Ratiometric Comparison of the PH-Akt-GFP and Lyn-mCherry localization. The PH-Akt-GFP/Lyn-mCherry ratio image sequence is shown for a polarized cell on pattern, before and after PDGF stimulation. The GFP/mCherry ratio had a relatively even profile before stimulation. After stimulation, the ratio image showed a small value (blue) at the center of cell body, a intermediate value (green) at two lateral sides of the patterned region and a large value (red) in membrane ruffles (middle panel, white arrow) and the protrusion region (bottom panel, white arrow head), indicating that PH-Akt-GFP had a more significant presence in the ruffles and protrusion than Lyn-mCherry. Scale bar: 10 µm. (TIF) [file pone.0021293.s001.tif]

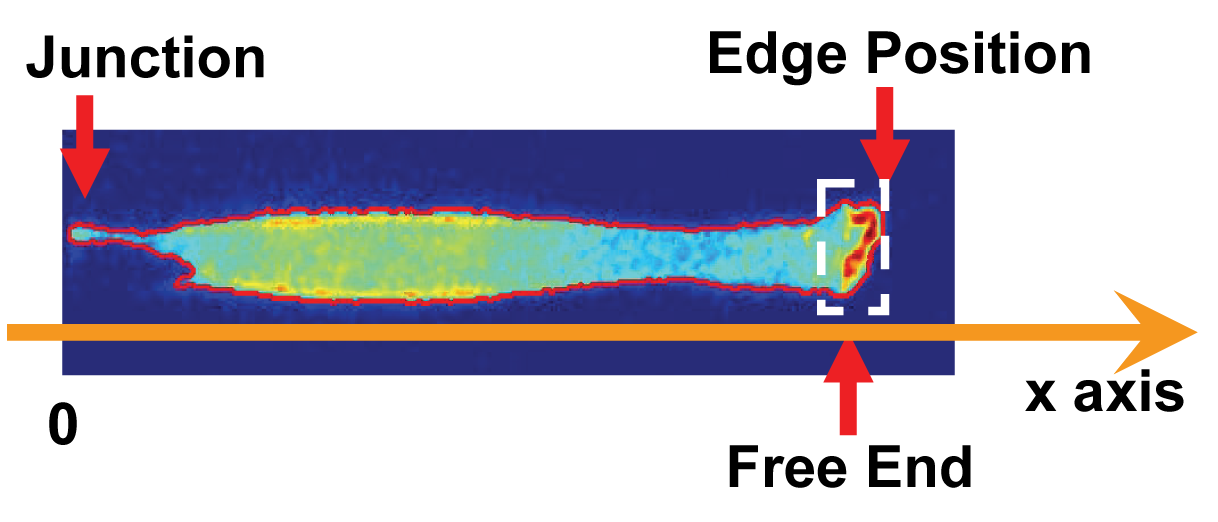

Supplement: Figure S2 — The edge position at the free end. After the fluorescent image of a cell is rotated and aligned along the x-axis with its junction to a neighboring cell at the left side, the edge position of the free end (red arrow) is calculated based on the maximal x-pixel number of the detected cell mask (outlined in red). The free end region is chosen as the 1/10 of cell length at the free end, as outlined in the white and dashed rectangle. (TIF) [file pone.0021293.s002.tif]

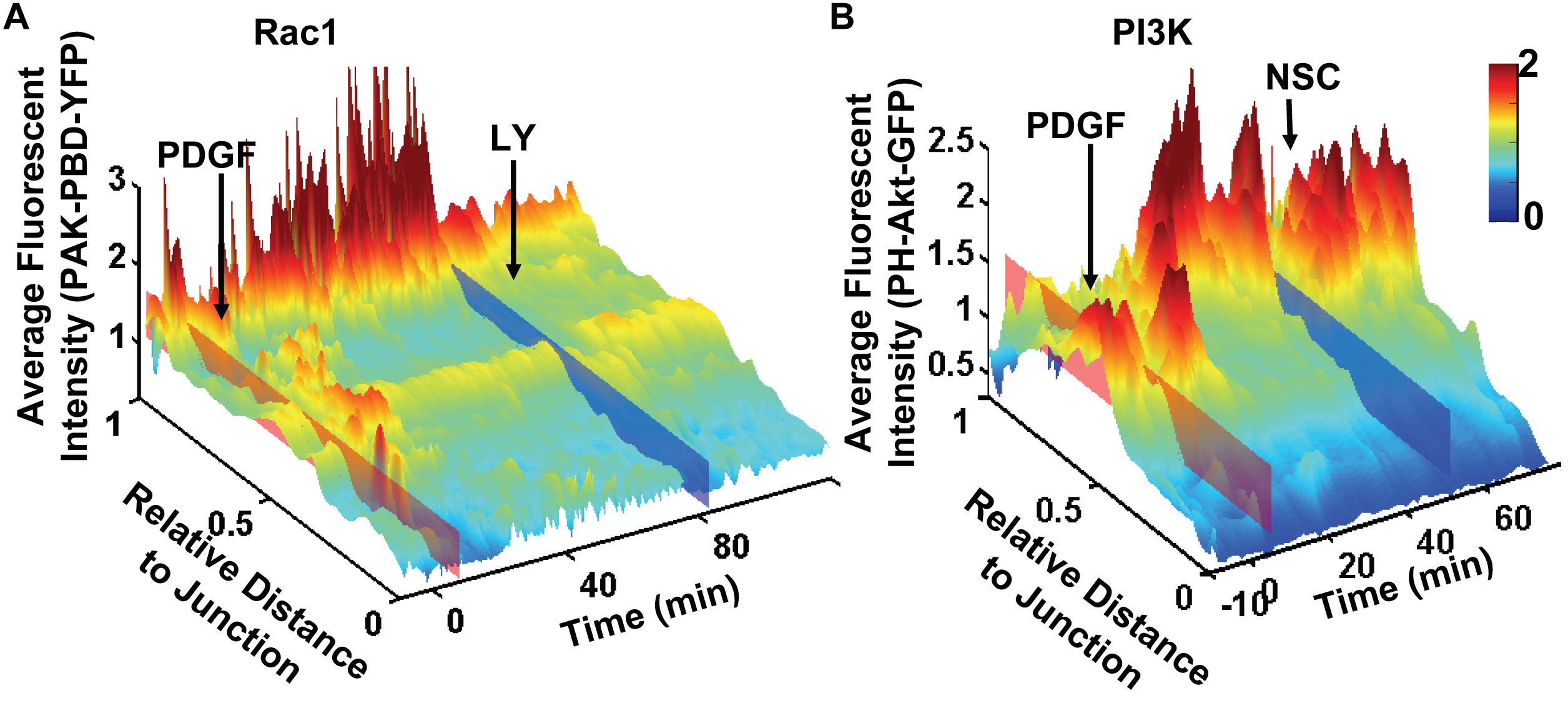

Supplement: Figure S3 — The effect of inhibitors. (A) The Rac1 activity landscape of an MEF treated with PDGF (0 min, red plane) prior to PI3K inhibitor (LY) application (∼80 min, blue plane) is shown as a function of time and the distance to the junction. (B) The PI3K activity landscape of an MEF treated with PDGF (0 min, red plane) prior to Rac1 inhibitor (NSC) application (∼50 min, blue plane) is shown as a function of time and the distance to the junction. (TIF) [file pone.0021293.s003.tif]

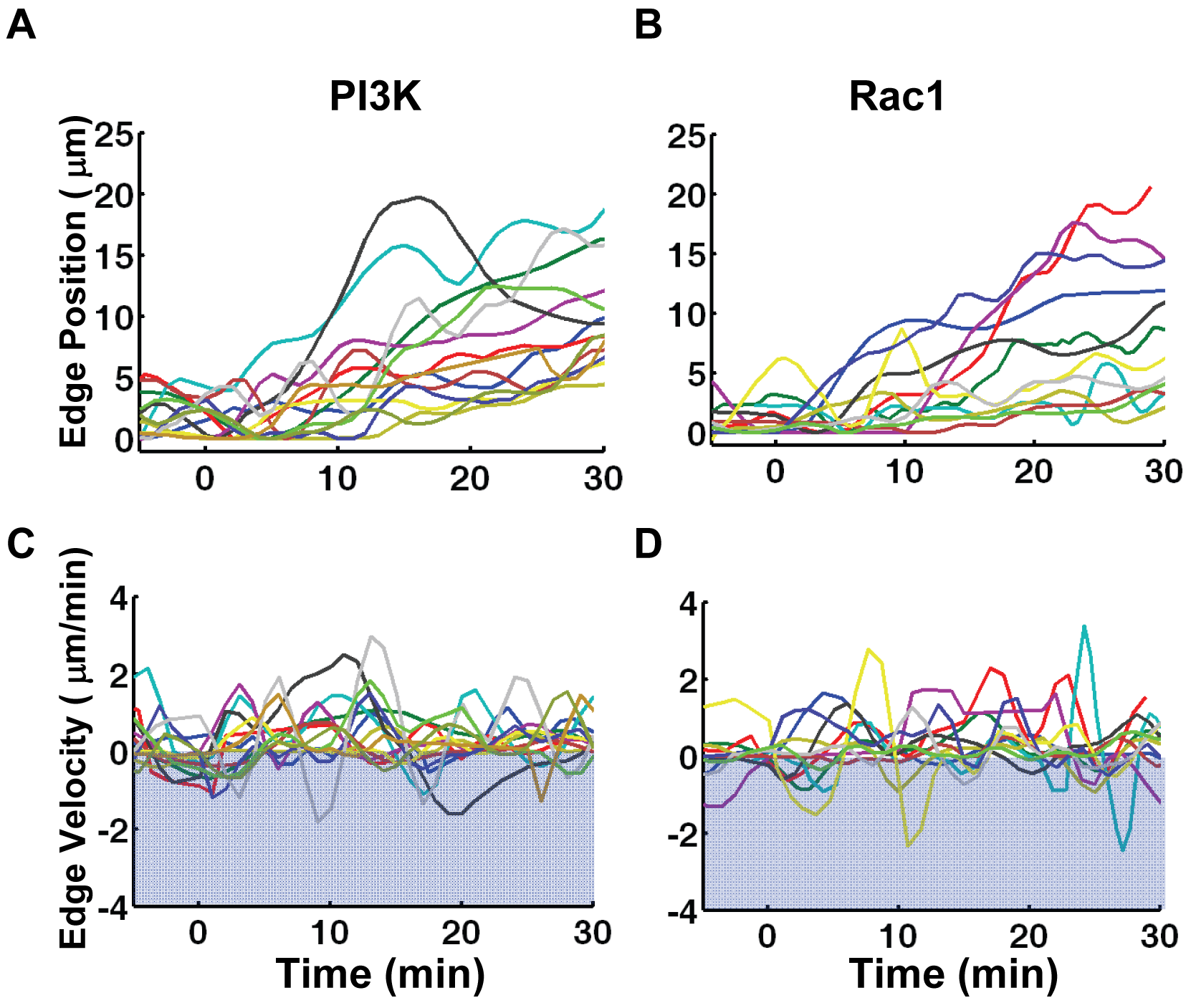

Supplement: Figure S4 — The expression of different fluorescent probes did not affect the front-edge dynamics. (A) and (B), the smoothed time courses of the front edge position in the cells with PI3K or Rac1 probes respectively, with each line representing one cell; (C) and (D), the smoothed time courses of the front edge velocity in the cells with PI3K or Rac1 probes respectively, with each line representing one cell. The shaded regions indicate negative velocity. (TIF) [file pone.0021293.s004.tif]

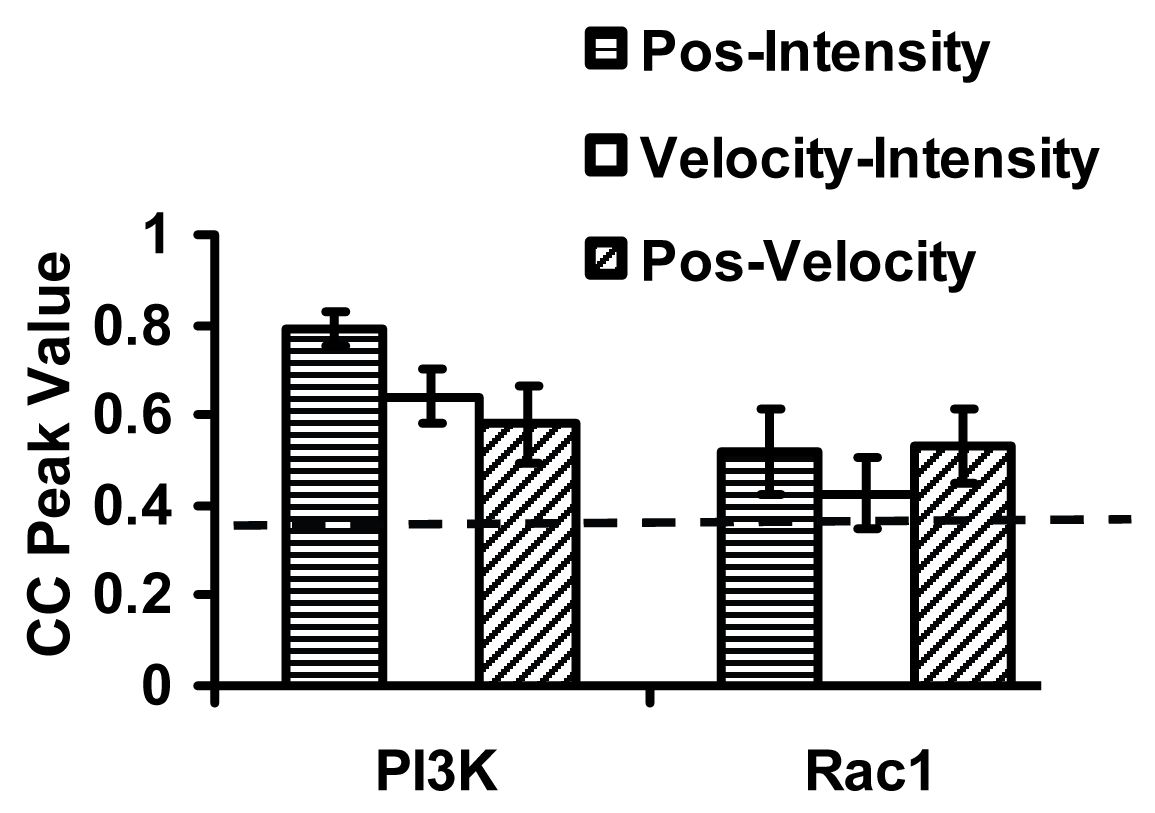

Supplement: Figure S5 — The cross-correlation peak values. The mean and standard error of means (SEM) of the peak values of the cross-correlation curves are shown for PI3K and Rac1 activities. (TIF) [file pone.0021293.s005.tif]

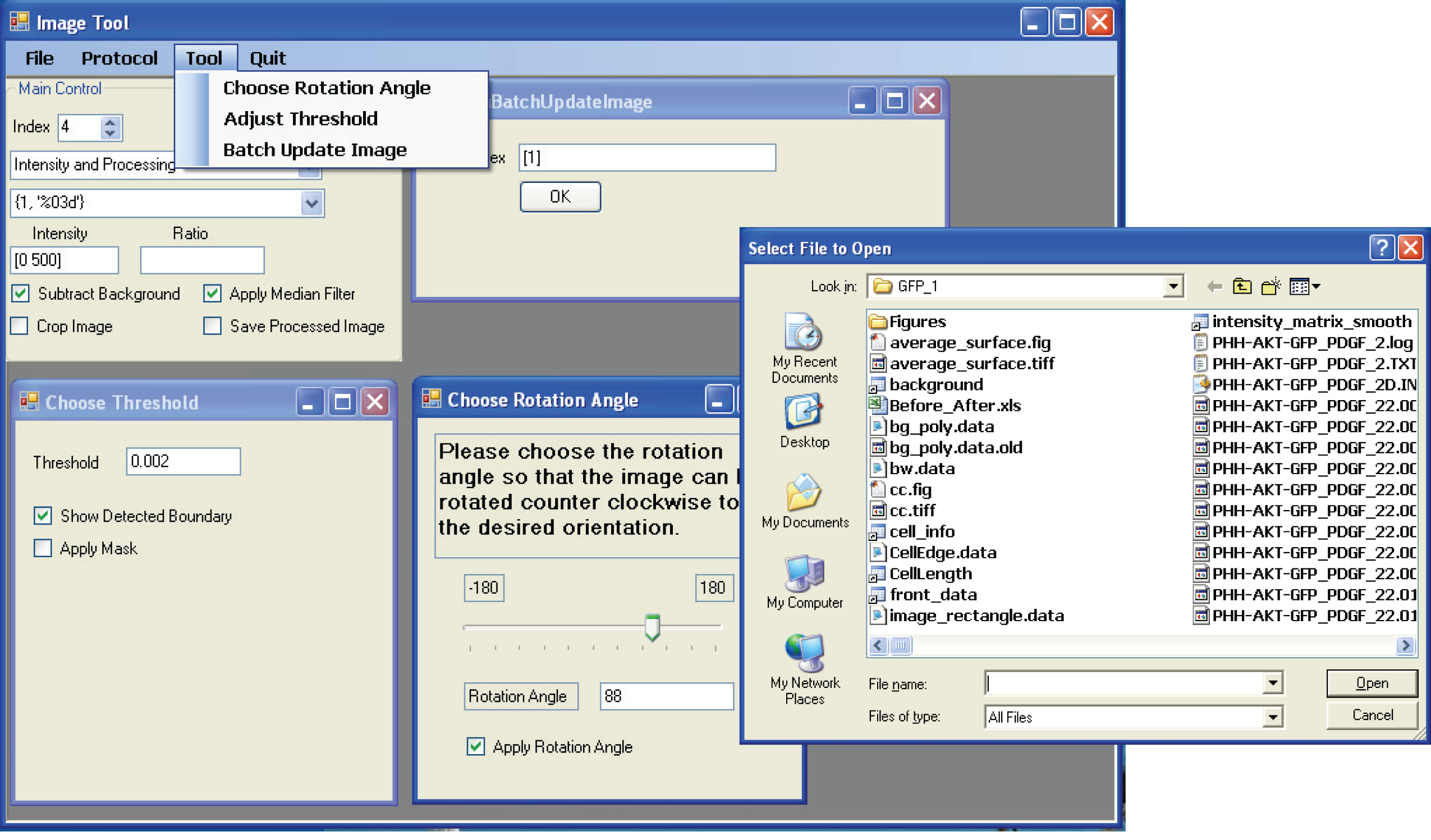

Supplement: Figure S6 — The graphic user interface (GUI). The GUI was implemented in Visual Basic. It allows the users to display, filter, rotate and crop images, and adjust the rotation angle and threshold value of imaging segmentation for each cell. (TIF) [file pone.0021293.s006.tif]

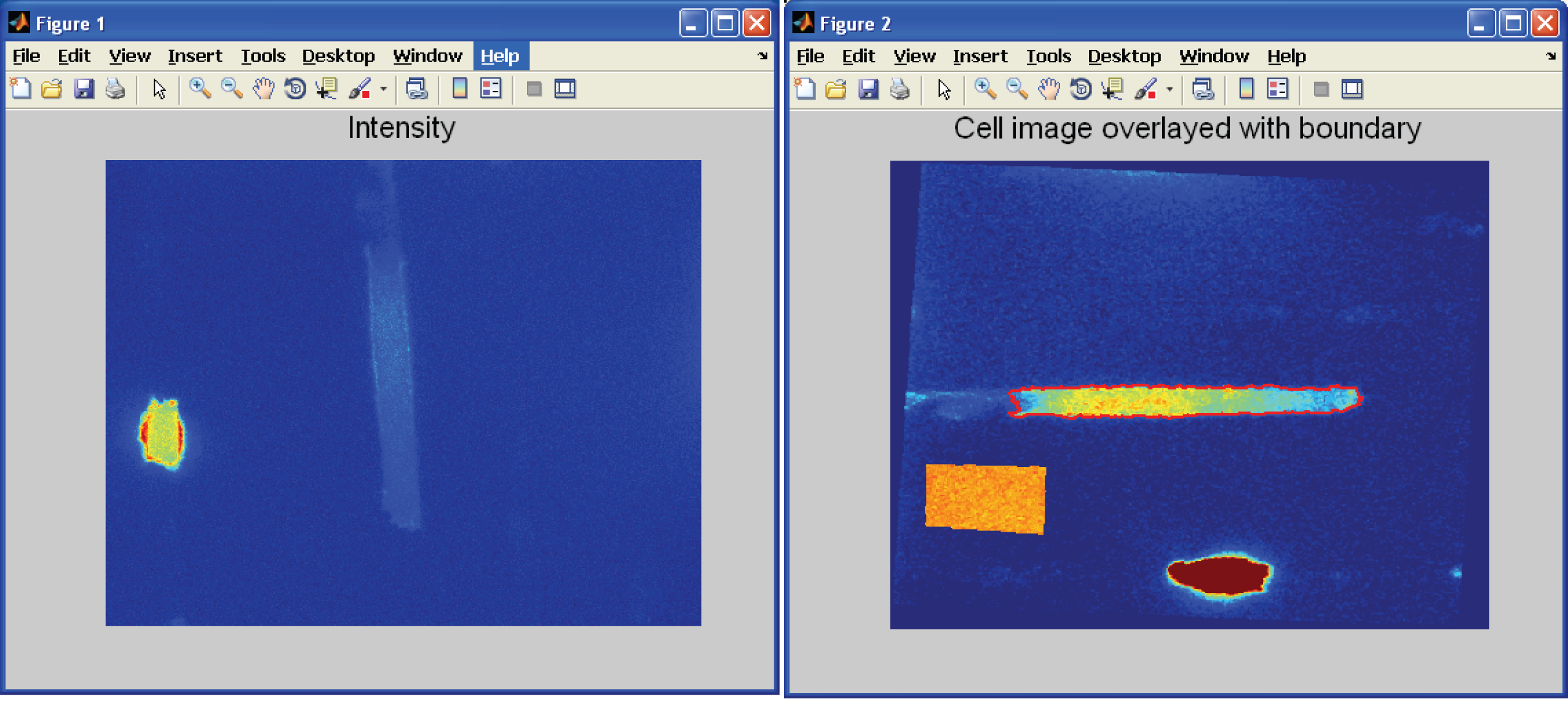

Supplement: Figure S7 — Image pre-processing with GUI. Left: the raw data image shown in pseudo color. Right: the raw image was processed by a medium filter with a window size of 3×3 pixels, background-subtracted, intensity-scaled, and rotated so that the free end of the cell was aligned along the positive direction of the x-axis. The image segmentation threshold was also selected and the boundary of the cell mask calculated and shown in solid red. The orange block on the lower left corner of the image highlights the area where the background signal was calculated for subtraction. (TIF) [file pone.0021293.s007.tif]
